# Outstanding Photoluminescence in Pr^3+^-Doped Perovskite Ceramics

**DOI:** 10.3390/mi9090419

**Published:** 2018-08-21

**Authors:** Jiameng Zhang, Yanan Hao, Meihua Bi, Guoyan Dong, Xiaoming Liu, Ke Bi

**Affiliations:** 1 State Key Laboratory of Information Photonics and Optical Communications, School of Science, Beijing University of Posts and Telecommunications, Beijing 100876, China; zhangjiameng@bupt.edu.cn (J.Z.); bimeihua@bupt.edu.cn (M.B.); 2 College of Materials Science and Opto-Electronic Technology, University of Chinese Academy of Sciences, Beijing 100049, China; 3 Key Laboratory of Electromagnetic Processing of Materials (Ministry of Education), Northeastern University, Shenyang 110819, China; liuxm@smm.neu.edu.cn; 4 Department of New Energy Science & Engineering, School of Metallurgy, Northeastern University, Shenyang 110819, China

**Keywords:** perovskite ceramics, Pr^3+^-doped, solid-state reactions, photoluminescence

## Abstract

Ba (Zr_0.2_Ti_0.8_) O_3_-50% (Ba_0.7_Ca_0.3_) TiO_3_ (BZT-0.5BCT) ceramics with different doping contents of Pr^3+^ were prepared by the conventional solid-state reaction. The phase structure and crystallinity of the fabricated ceramics were investigated by X-ray diffraction, Raman spectroscopy, and scanning electron microscopy. Photoluminescence (PL) emission spectra were measured to analyze the PL characteristics. The strong intensities of a green band at 489 nm and a red band at 610 nm were observed. The maximum emission intensity of the PL spectrum was achieved in the BZT-0.5BCT ceramic with 0.2% mol of Pr^3+^ ions. Furthermore, the PL spectra of BZT-0.5BCT ceramics were found to be sensitive to polarization of the ferroelectric ceramics. Compared with the unpoled ceramics, the green emission increased about 42% and a new emission peak at 430 nm appeared for the poled ceramics. With excellent intrinsic ferroelectricity and an enhanced PL property, such material has potential to realize multifunctionality in a wide application range.

## 1. Introduction

In recent years, perovskite materials have attracted wide attention for their excellent physical properties, such as piezoelectricity, ferroelectricity, and ferromagnetism [1,2,3,4]. Owing to the adjustable crystal structure, rare-earth doping is widely used to realize novel or multifunctionality in perovskite materials [5,6]. Especially, the photoluminescence (PL) behavior of rare-earth-doped perovskite material is extensively studied for its wide range of applications [7,8,9,10,11]. The principle of PL emission in such a material is ascribed to the energy transfer from the host materials to rare earth ions [12,13,14,15]. The resulting materials show multifunctional properties, which can combine the PL property with excellent ferroelectricity or dielectric properties in one material, and therefore possess high potential for coupling devices, sensors, and other multifunctional applications.

It is well-known that (Ba, Ca) TiO_3_ (abbreviated as BCT) is a typical perovskite material [16,17,18]. In order to explore the potential for multifunctional applications of BCT ceramics, some new strategies were taken to improve the PL, ferroelectric, and piezoelectric properties of BCT ceramics. For instance, recently, Zou et al. found that the red emission of Pr^3+^-doped BaTiO_3_-CaTiO_3_ ceramics was enhanced greatly by poling [19]. Jia et al. found that the PL intensity of Pr^3+^-doped (Bi_0.5_Na_0.5_) TiO_3_ ceramics could be enhanced by ferroelectrics remnant polarization [20]. Also, some researchers found that the ferroelectric and piezoelectric properties of BCT ceramics can be greatly enhanced by doping an amount of Zr^4+^. For instance, Zhang et al. obtained a piezoelectric constant *d*_33_ as high as 200 pC/N by introducing Zr^4+^ dopant in (Ca, Ba) TiO_3_ [21]. Zhang et al. reported (Ba, Ca) (Ti, Zr) O_3_ (abbreviated as BCTZ) ceramics which have high piezolelectric and ferroelectric properties [22]. Liu et al. reported a BCTZ ceramic which has a high piezoelectric constant *d*_33_ (~620 pC/N) [23]. However, there are few surveys on the improvement of PL properties of BCTZ-based ceramics.

In the current work, we choose BZT-0.5BCT as the target material, which was reported to possess superior piezoelectricity and ferroelectricity properties [23]. Pr^3+^-doped BZT-0.5BCT ceramics have been successfully synthesized by the conventional solid-state reaction method. The crystal structure, morphology, and substitution mechanism were thoroughly investigated, and the related mechanism has been discussed. Meanwhile, the PL properties of poled and unpoled samples are measured, which shows that the PL property of the Pr^3+^-doped BZT-0.5BCT ceramics is enhanced by poling. The characteristics suggest that the Pr^3+^-doped BZT-0.5BCT ceramics are promising multifunctional materials.

## 2. Materials and Methods

BZT-0.5BCT-*x*Pr_6_O_11_ ceramics (*x* = 0%, 0.1%, 0.2%, 0.3%) were prepared by conventional solid-state reaction. BaCO_3_ (99%), CaCO_3_ (99%), TiO_2_ (99%), ZrO_2_ (99%), and Pr_6_O_11_ (99%) powders were used as raw materials, which were mixed according to a predetermined ratio in deionized water media and ball-milled for 8 h. The well-mixed raw materials were then dried and calcined at 1150 °C for 2 h. Thereafter, they were remixed and pressed into disk-shaped pellets 10 mm in diameter. Finally, these pellets were placed in zirconia crucible, and heated to 1450 °C with a heating rate of 100 °C/h and held for 2 h. The phase structure of the ceramics was determined by X-ray diffraction analysis (XRD; D/Max-2500, Rigaku Co., Tokyo, Japan). The microstructures were observed by using a scanning electron microscope (SEM; JSM-7001F, JEOL Ltd., Tokyo, Japan). Raman spectra were recorded by using 633-nm excitation sources and a micro-Raman spectrometer at different temperatures (RM2000, Renishaw Co., Wotton-under-Edge, UK). The PL spectra were recorded by using a spectrophotometer under the excitation of a 362-nm laser diode (FLS920 Edinburgh, Livingston, UK). For polling, silver electrodes were prepared on both sides of the sintered pellets. Then, these pellets were subjected to a 25 kV/cm electric field for 30 min. After that, the electrodes were polished away for the PL spectra measurement and the surface roughness of the unpoled and poled samples was kept consistent.

## 3. Results and Discussion

**Figure 1:**
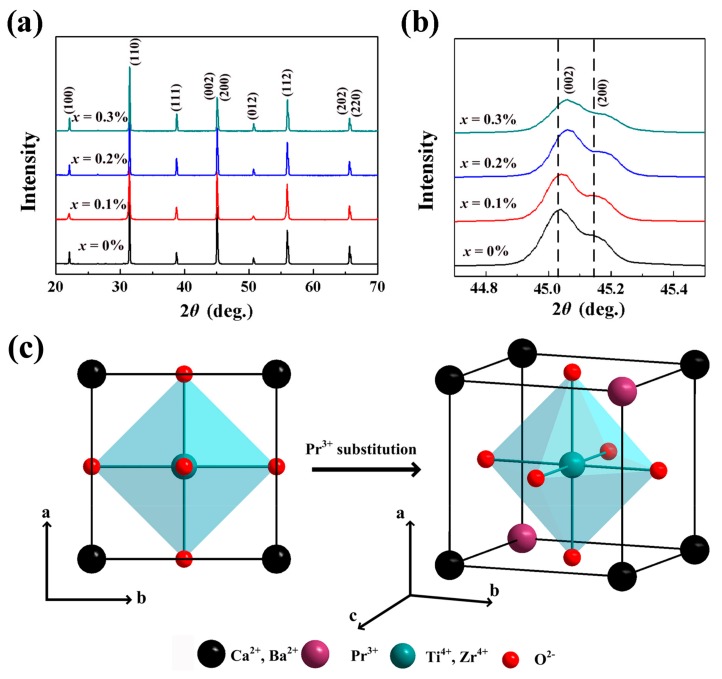
(**a**) XRD patterns and (**b**) (002) and (200) diffraction peaks for the as-obtained BZT-0.5BCT-*x*Pr^3+^ ceramics; (**c**) Schematic illustration for the crystal structure of Pr^3+^-doped BZT-0.5BCT.

**Figure 2:**
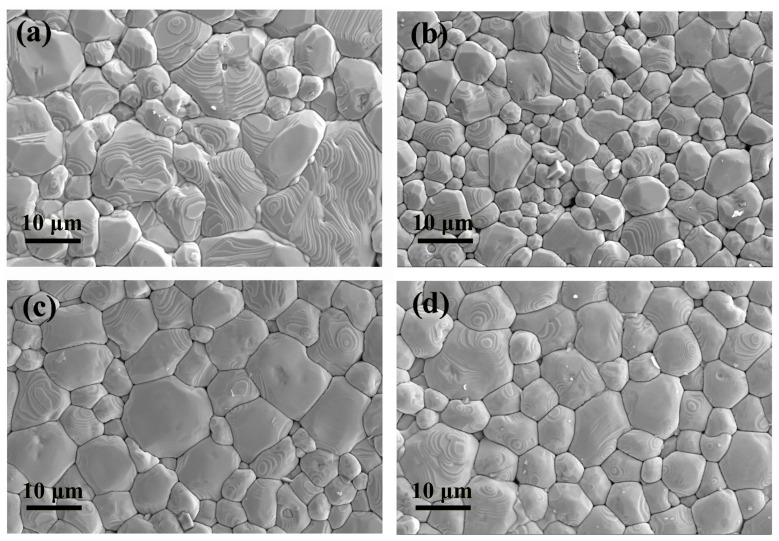
SEM micrographs of the BZT-0.5BCT-*x*Pr^3+^ ceramics: (**a**) *x* = 0; (**b**) *x* = 0.1; (**c**) *x* = 0.2; (**d**) *x* = 0.3.

The X-ray diffraction patterns of BZT-0.5BCT with different doping contents of Pr^3+^ are shown in Figure 1a. It is obvious that all the ceramics are in orthorhombic phase and there are no additional peaks that related to rare-earth oxides. These results are consistent with previous reports and confirm that the rare-earth ions have been successfully doped into the crystal lattices of the ceramics [24,25,26]. Generally speaking, the phase structure of BCTZ-based ceramics could be determined by the (002) and (200) diffraction peaks at 2*θ* ≈ 44–46°. The enlarged (002) and (200) peaks for the as-obtained ceramics are shown in Figure 1b. It is found that when Pr^3+^ was doped into BZT-0.5BCT ceramics, the XRD peaks shift slightly toward a higher angle, implying the reduction of lattice parameters with Pr^3+^ doping. As is known, a rare earth ion may occupy different sites of the ceramic lattice depending on its ionic radius and charge [27]. The value of the Pr^3+^ (*r* = 1.13 Å) radius lies between the values of the Ca^2+^ (*r* = 1.12 Å) and Ba^2+^ (*r* = 1.42 Å) radius. So, it is estimated that Pr^3+^ ions substitute the Ba^2+^ sites according to the XRD measurement results. Based on the XRD results, the schematic illustration for the crystal structure of Pr^3+^-doped BZT-0.5BCT ceramics can be described as Figure 1c. SEM images of the ceramics with different doping contents of Pr^3+^ are shown in Figure 2. Derived from Figure 2a, the grain sizes of BZT-0.5BCT ceramics without Pr^3+^ ions were about 9–11 µm. As shown in Figure 2b–d, the average grain sizes of BZT-0.5BCT ceramics with different doping contents of Pr^3+^ were around 7 µm. It is obvious that these ceramics have high compact density and show an uneven grain size distribution.

**Figure 3:**
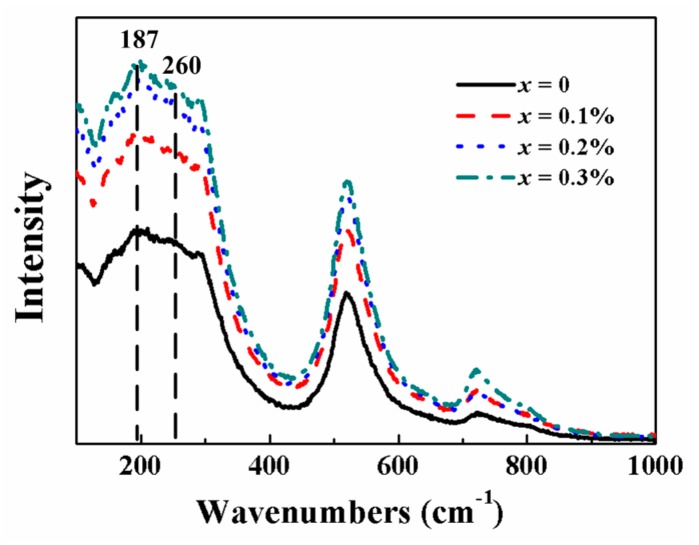
Raman spectra of the BZT-0.5BCT-*x*Pr_6_O_11_ ceramics measured at room temperature under the 633-nm source excitation.

To further investigate the crystal structure of BZT-0.5BCT ceramics, we measured the Raman spectra of the samples. Figure 3 shows the Raman spectra of BZT-0.5BCT ceramics with different doping contents of Pr^3+^ at room temperature within the wavenumber range of 100–1000 cm^−1^, and the excited laser source is 633 nm. We can see that both the ceramics with and without Pr^3+^ show typical Raman spectra of BZT-0.5BCT with a perovskite structure [28]. The Raman band (~260 cm^−1^) represents a double-degenerate O-Ti, Zr-O stretching vibration, and was sensitive to the change of phase structure. We can see that the position of the band has no obvious shift, and the intensity increased after Pr^3+^ was doped into BZT-0.5BCT ceramics. This phenomenon indicated that the vibration of O-Ti, Zr-O was affected by the addition of Pr^3+^. Meanwhile, it is obvious that there are no new peaks in the Raman spectra of the ceramic with different doping contents of Pr^3+^, implying that the introduction of Pr^3+^ may not bring about additional modes. The results agreed well with the XRD results of BZT-0.5BCT ceramics with different doping contents of Pr^3+^.

**Figure 4:**
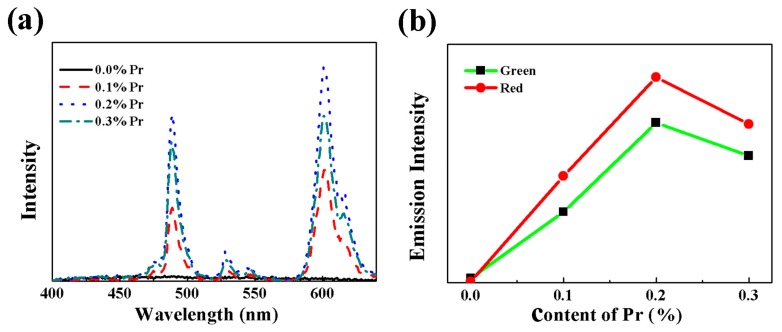
(**a**) Photoluminescence (PL) spectra of the BZT-0.5BCT-*x*Pr_6_O_11_ ceramics under 362 nm excitation; (**b**) Plotted curve of the green and red emission intensities for different *x* under 362 nm excitation.

In order to explore the effect of the content of Pr^3+^ on PL properties, the PL spectra of the BZT-0.5BCT ceramics with different doping contents of Pr^3+^ were measured under infrared radiation excitation of 362 nm and are shown as Figure 4a. We can see that the BZT-0.5BCT ceramic without Pr^3+^ has no emission. However, two PL bands centered at 610 nm and 489 nm can be observed for the BZT-0.5BCT ceramics with different doping contents of Pr^3+^. The strong visible red emission at 610 nm was ascribed to Pr^3+^ ^1^D_2_ to ^3^H_4_ emission. In addition, the green emission at 489 nm was ascribed to Pr^3+ 3^P_0_ to ^3^H_4_ emission. The PL emission peaks of the BZT-0.5BCT-*x*Pr^3+^ ceramics are consistent with the PL process of Pr^3+^ as indicated in previous literature [28]. Meanwhile, the emission peaks have no obvious shift for the ceramics with different doping contents of Pr^3+^. Also, it is observed that the intensities for the green emission band and the red emission band are firstly enhanced as the doping content of Pr^3+^ increased. However, the PL intensities suddenly drop when the doping content of Pr^3+^ is higher than 0.2% as shown in Figure 4b. The BZT-0.5BCT ceramic with Pr^3+^-doping content of 0.2% exhibits the most excellent PL property. According to previous studies, the presence of the inflection point was probably ascribed to the existence of a concentration-quenching effect [29,30,31,32]. The concentration-quenching effect is mainly caused by the energy transfer among Pr^3+^ ions. As the content of Pr^3+^ increases, the distance between Pr^3+^ becomes smaller and the probability of energy transfer between Pr^3+^ increases. Finally, the energy loss caused by energy transfer among Pr^3+^ increases and the transition energy of Pr^3+^ is accordingly decreased. Therefore, the PL intensities drop when the Pr concentration is beyond *x* = 0.02.

**Figure 5:**
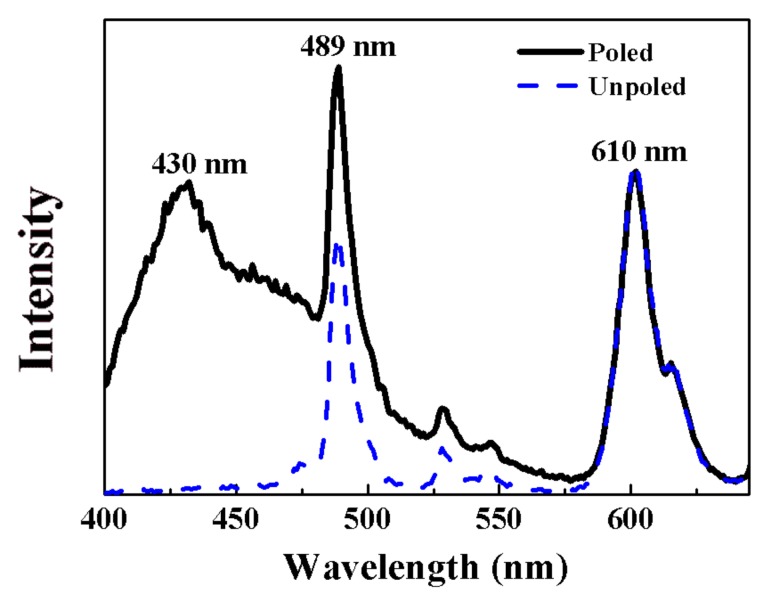
PL spectra of poled and unpoled BZT-0.5BCT ceramics with 0.2% Pr^3+^ under 362 nm excitation.

In order to enlarge the emission intensity of the BZT-0.5BCT ceramics doped with 0.2% Pr^3+^ ions, we polarized the ceramics and measured the PL spectra of poled and unpoled BZT-0.5BCT-0.2% Pr^3+^ ceramics under normal temperature with an excitation laser source of 362 nm as shown in Figure 5. Compared with the unpoled samples, the red emission at 610 nm has no obvious variation for the poled ceramics. However, the emission intensity of the green band at 489 nm increased about 42%. Besides this, a strong emission peak at 430 nm appeared, which is unusual for the BZT-0.5BCT ceramics. Also, it is worth noting that the position of both the green band centered at 489 nm and the red band centered at 610 nm do not shift. This phenomenon is attributed to the emission spectra of Pr^3+^ being sensitive to the variation of the environment in the BZT-0.5BCT ceramics. After poling, the polarization will align along the direction of the external electric field and the local electric field around Pr^3+^ site will emerge. Therefore, the crystalline field of the Pr^3+^ ions would be changed because of the change of local electric field. So, the polarization of the ceramics is the key factor that is responsible for the change of green emission and the appearance of a new peak at 430 nm. Also, a related report presents that the emission intensity from rare earth elements increases with the intensity of the excitation light [33]. The as-synthesized Pr^3+^-doped BZT-0.5BCT ceramics show great potential for pursing novel coupled properties in the application of multifunctional materials.

## 4. Conclusions

In conclusion, the Pr^3+^-doped BZT-0.5BCT ceramics were prepared via a conventional solid-state reaction method. The XRD analyses and Raman spectra confirmed that Pr^3+^ ions were doped into BZT-0.5BCT host successfully and occupied the A site in the perovskite structure of BZT-0.5BCT. The SEM images reveal the dense and uniform microstructure of the ceramics. The PL emission spectra of the BZT-0.5BCT ceramics with different doping contents of Pr^3+^ show a typical red emission band at 610 nm and a green emission band at 489 nm due to the energy level transition of Pr^3+^. Additionally, the maximum emission intensity is obtained by the BZT-0.5BCT ceramic doped with 0.2% Pr^3+^. Moreover, after poling at 25 kV/m, the BZT-0.5BCT ceramics show a greatly enhanced PL property. Compared with the unpoled ceramics, the emission intensity of the green band increases about 42% and a new band at 430 nm emerged. By improving the PL property effectively in BCTZ ceramics, this study provides a promising multifunctional material for future applications.

## References

[1] Park K.I., Xu S., Liu Y., Hwang G.T., Kang S.J., Wang Z.L., Lee K.J. (2010). Piezoelectric BaTiO_3_ thin film nanogenerator on plastic substrates. Nano Lett..

[2] Bi K., Bi M.H., Hao Y.N., Luo W., Cai Z.M., Wang X.H., Huang Y.H. (2018). Ultrafine core-shell BaTiO_3_@SiO_2_ structures for nanocomposite capacitors with high energy density. Nano Energy.

[3] Scott J.F. (2007). Applications of modern ferroelectrics. Science.

[4] Bi M.H., Zhang J.M., Hao Y.N., Lei M., Bi K. (2017). Particle size effect of BaTiO_3_ nanofillers on the energy storage performance of polymer nanocomposites. Nanoscale.

[5] Ishihara T., Matsuda H., Takita Y. (1995). ChemInform abstract: Doped PrMnO_3_ perovskite oxide as a new cathode of solid oxide fuel cells for low temperature operation. J. Electrochem. Soc..

[6] Xie P., Wang Z., Zhang Z., Fan R.H., Cheng C.B., Liu H., Liu Y., Li T.X., Yan C., Wang N. (2018). Silica microsphere templated self-assembly of a three-dimensional carbon network with stable radio-frequency negative permittivity and low dielectric loss. J. Mater. Chem. C.

[7] Okamoto S., Kobayashi H., Yamamoto H. (1999). Enhancement of characteristic red emission from SrTiO_3_:Pr^3+^ by Al addition. J. Appl. Phys..

[8] Wang X., Xu C.N.H. (2005). Yamada, Enhancement of Photoluminescence in CaTiO_3_:Pr^3+^ by Ba and Sr Substitution for Ca. Jpn. J. Appl. Phys..

[9] Li J., Chai X., Peng D.F., Zou H., Wang X. (2014). Largely enhanced electromechanical properties of BaTiO_3_-(Na_0.5_Er_0.5_) TiO_3_ lead-free piezoelectric ceramics. Appl. Phys. Lett..

[10] Wu X., Kwok K.W. (2014). Mid-IR to Visible Photoluminescence, Dielectric, and Ferroelectric Properties of Er-Doped KNLN Ceramic. J. Am. Ceram. Soc..

[11] Zhao Y., Ge Y., Zhang X., Zhao Y.Z., Zhou H., Li J., Jin H.B. (2016). Effect of phase structure changes on the lead-free Er^3+^-doped (K_0.52_Na_0.48_)1-xLixNbO_3_. J. Alloys Compd..

[12] Zhang J.M., Hao Y.N., Wang Q.M., Xu J.C., Guo L.M., Bi K. (2018). Enhanced photoluminescence properties of SrTiO_3_:Pr^3+^ nanocrystals by the “TEG-sol” method. APL Mater..

[13] Zhang J.C., Long Y.Z., Wang X., Xu C.N. (2014). Controlling elastico-mechanoluminescence in diphase (Ba, Ca) TiO_3_:Pr^3+^ by co-doping different rare earth ions. RSC Adv..

[14] Boutinaud P., Pinel E., Dubois M., Vink A.P., Mahiou R. (2005). UV-to-red relaxation pathways in CaTiO_3_:Pr^3+^. J. Lumin..

[15] Jia W., Jia D., Rodriguez T. (2006). UV excitation and trapping centers in CaTiO_3_:Pr^3+^. J. Lumin..

[16] Feng Z.P., Hao Y.N., Bi M.H., Bi K. (2018). Highly dispersive Ba_0.5_Sr_0.5_TiO_3_ nanoparticles modified P(VDF-HFP)/PMMA composite films with improved energy storage density and efficiency. IET Nanodielectr..

[17] Rao R., Roy A.P., Dasannacharya B.A. (1996). Raman study of phase transition in ferroelectric Ba_0.95_Ca_0.05_TiO_3_. Pramana.

[18] Hao Y.N., Wang X., Bi K., Zhang J.M., Huang Y., Wu L., Zhao P., Xu K., Lei M., Li L. (2017). Significantly enhanced energy storage performance promoted by ultimate sized ferroelectric BaTiO_3_ fillers in nanocomposite films. Nano Energy.

[19] Zou H., Peng D., Wu G. (2013). Polarization-induced enhancement of photoluminescence in Pr^3+^ doped ferroelectric diphase BaTiO_3_-CaTiO_3_ ceramics. J. Appl. Phys..

[20] Tian X., Wu Z., Jia Y. (2013). Remanent-polarization-induced enhancement of photoluminescence in Pr^3+^-doped lead-free ferroelectric (Bi_0.5_Na_0.5_) TiO_3_ ceramic. Appl. Phys. Lett..

[21] Zhang S.W., Zhang H., Zhang B.P. (2009). Dielectric and piezoelectric properties of (Ba_0.95_Ca_0.05_) (Ti_0.88_Zr_0.12_) O_3_. J. Eur. Ceram. Soc..

[22] Zhang Y., Glaum J., Groh C., Ehmke M.C., Blendell J.E., Bowman K.J., Hoffman M.J. (2014). Correlation between piezoelectric properties and phase coexistence in (Ba, Ca) (Ti, Zr) O_3_. J. Am. Ceram. Soc..

[23] Liu W., Ren X. (2009). Large piezoelectric effect in Pb-free ceramics. Phys. Rev. Lett..

[24] Kyômen T., Sakamoto R., Sakamoto N. (2005). Photoluminescence Properties of Pr-Doped (Ca, Sr, Ba) TiO_3_. Chem. Mater..

[25] Li W., Xu Z., Chu R. (2011). High piezoelectric d_33_, coefficient of lead-free (Ba_0.93_Ca_0.07_) (Ti_0.95_Zr_0.05_) O_3_, ceramics sintered at optimal temperature. Mater. Sci. Eng. B.

[26] Zhang L., Wang X., Liu H. (2010). Structural and Dielectric Properties of BaTiO_3_-CaTiO_3_-SrTiO_3_ Ternary System Ceramics. J. Am. Ceram. Soc..

[27] Ryu H., Singh B.K., Bartwal K.S. (2008). Novel efficient phosphors on the base of Mg and Zn co-doped SrTiO_3_:Pr. Acta Mater..

[28] Zhang P., Shen M., Fang L. (2008). Pr^3+^ photoluminescence in ferroelectric (Ba_0.77_Ca_0.23_) TiO_3_ ceramics: Sensitive to polarization and phase transitions. Appl. Phys. Lett..

[29] Xie R.J., Hirosaki N., Sakuma K., Yamamoto Y., Mitomo M. (2004). Eu^2+^-doped Ca-α-SiAlON: A yellow phosphor for white light-emitting diodes. Appl. Phys. Lett..

[30] Wang X., Deng X., Wen H. (2006). Phase transition and high dielectric constant of bulk dense nanograin barium titanate ceramics. Appl. Phys. Lett..

[31] Qiu J., Miura K., Sugimoto N., Hirao K. (1997). Preparation and fluorescence properties of fluoroaluminate glasses containing Eu^2+^ ions. J. Non-Cryst. Solids.

[32] He X., Dong W., Zheng F. (2010). Effect of tartaric acid on the microstructure and photoluminescence of SrTiO_3_:Pr_3+_, phosphors prepared by a sol-gel method. Mater. Chem. Phys..

[33] Cai H.L., Wu X.S., Gao J. (2009). Effect of oxygen content on structural and transport properties in SrTiO_3− x_, thin films. Chem. Phys. Lett..

